# Extrapelvic endometriosis located individually in the rectus abdominis muscle: a rare cause of chronic pelvic pain (a case report)

**DOI:** 10.11604/pamj.2022.42.242.36325

**Published:** 2022-07-29

**Authors:** Anna Thanasa, Efthymia Thanasa, Evangelos Kamaretsos, Evangelos-Ektoras Gerokostas, Ioannis Thanasas

**Affiliations:** 1 Department of Health Sciences, Medical School, Aristotle University of Thessaloniki, Thessaloniki, Greece,; 2 Department of Obstetrics and Gynecology of General Hospital in Trikala, Trikala, Greece

**Keywords:** Rectus abdominis muscles, caesarean section, diagnosis, treatment, case report

## Abstract

Endometriosis of the rectus abdominis muscle is an extremely rare form of extrapelvic localization of the disease. It is usually iatrogenic and develops after caesarean section or gynecological surgery. Preoperative diagnosis is very difficult and a challenge for gynecologists and surgeons; thus, the diagnosis is histological. The treatment of choice consists of wide local excision of the lesion on healthy margins. We cite a case of isolated endometriosis in the rectus abdominis muscles in a 46-year-old patient with a previous caesarean section, the diagnosis of which was made randomly when performing abdominal total hysterectomy for the treatment of chronic pelvic pain. Histological examination of the surgical specimen confirmed the diagnosis. Simultaneously, the surgical specimen of the uterus and ovaries was free of endometriosis. Postoperatively, the patient mentioned discharge of her symptoms. No further therapeutic intervention was deemed necessary, as it was considered that a complete resection of the endometrial tissue implantation from the muscles of abdominal wall was performed. The present case report lay emphasis on the significant difficulties involved in the preoperative diagnosis of endometriosis of the rectus abdominis muscle. Concurrently, it is pointed out that, despite its rarity, individual extrapelvic endometriosis located in the rectus abdominis muscle should be included among other pathological entities in the differential diagnosis of chronic pelvic pain in women of reproductive age, who gave birth by caesarean section or underwent gynecological surgery with abdominal or laparoscopic access.

## Introduction

Endometriosis is an invasive inflammatory non-neoplastic estrogen-dependent pathological condition, the main feature of which is the presence and development of ectopic functional endometrial tissue in areas outside the normal anatomical margins of the uterus [1]. It was probably first described by Rokitansky in the middle of the 19^th^ century [2]. Endometriosis is estimated to affect approximately 10% to 15% of women of reproductive age [3] and is more common in women with dysmenorrhea, dyspareunia and infertility [4]. It is usually located in the endopelvic organs and peritoneum. More rarely, it is possible to find extrapelvic implantation sites of the disease, such as in the abdominal wall, in the groin, in the vulva, in the navel in the episiotomy scar, in the urinary, gastrointestinal tract, respiratory system. In the few cases described in the literature to date, endometriosis can be isolated to the rectus abdominis muscles [5]. In this article, after the description of the case based on the literature, a brief review of this rare nosological entity is attempted.

## Patient and observation

**Patient information:** a 46-year-old reproductive patient, with a medical history of one caesarean section and a known presence of multiple and small uterine fibroids, visited the outpatient gynecological practice for pain in the lower abdomen for about ten years. The personal medical history of the patient was free. No problems were found from the urinary or gastrointestinal tract, to which chronic pelvic pain could be attributed. In addition to performing appendectomy at a young age, our patient had not undergone any other surgery.

**Clinical findings:** the patient described the onset of pain about 7 months after performing a caesarean section with a Pfannenstiel incision. Gradually, she reported a progressive deterioration of her condition. The pain described by the patient, initially as a feeling of heaviness in the lower abdomen, over the last one to two years has become of increasing intensity. Sometimes the pain may have been more intense during the days of menstruation, but usually the patient described non-intermittent pain in the lower abdomen of approximately the same intensity every day. The clinical examination did not reveal any palpable mass or other type of damage to the abdominal wall.

**Diagnostic assessment:** the findings from ultrasound scan, computerized tomography and magnetic resonance imaging of the abdomen were compatible with the presence of uterine fibroids. The levels of cancer antigen 125 in the blood serum were within the normal range.

**Figure 1:**
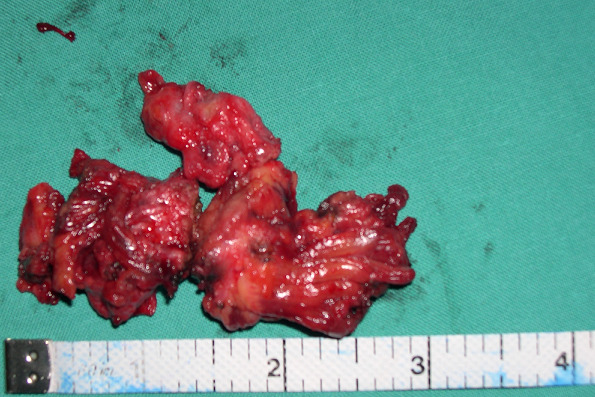
fibromuscular tissue blocks of the rectus abdominis muscle with endometriosis foci (our case)

**Figure 2:**
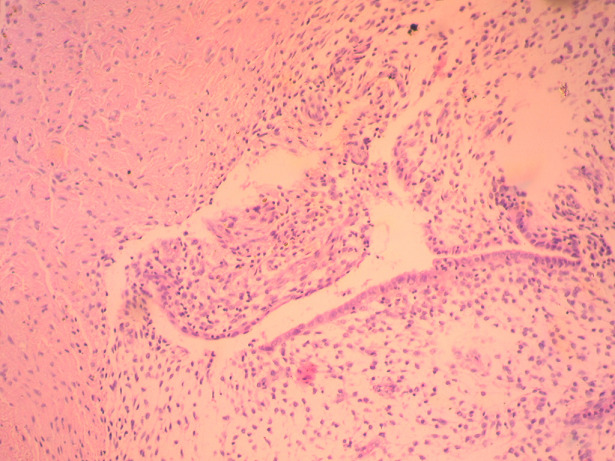
presence of an endometrial segment (glands and stroma) inside fibro-adipose tissue (our case)

**Figure 3:**
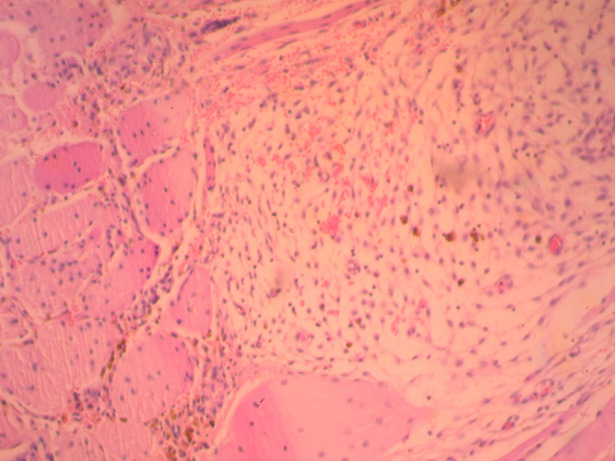
presence of endometrial stroma with hemosiderin-laden macrophages within skeletal muscle tissue (our case)

**Therapeutic intervention:** based on preoperative evaluation, chronic pelvic pain was attributed to the presence of uterine fibroids. After informing the patient and her relatives in more detail regarding the therapeutic approach of the disease, it was decided to perform abdominal total hysterectomy. Intraoperatively, in the rectus abdominis muscles and slightly above the level of the pubic symphysis, infiltration of the muscular wall was found by a hard-textured flat mass, in length 4-5 cm, the surface of which was solidly adhering to the parietal peritoneum. A wide resection of the lesion was performed, including bilaterally a widespread part of the wall of the rectus abdominis muscles and the matted peritoneum (Figure 1). The placement of surgical mesh was deemed necessary by the surgical team. Histological examination of the surgical specimen confirmed the diagnosis of endometriosis of the rectus abdominis muscles (Figure 2, Figure 3). Histologically, the surgical specimen of the uterus and ovaries was free of endometriosis. Excepting the small in size of the intramural and subserosal fibroids, the presence of adenomyosis, a large endometrial polyp or a submucosal leiomyoma of the uterus being prolapsed into the vagina was not detected.

**Follow-up and outcomes:** postoperatively, the patient mentioned relief of her symptoms. No further therapeutic pharmaceutical intervention was recommended, as it was considered that the endometriotic foci was completely resected from the abdominal wall.

**Informed consent**: this was sought and obtained from the patient. Anonymity was maintained for confidentiality.

## Discussion

Abdominal wall endometriosis is one of the rarest extrapelvic forms of endometrial disease. It is usually iatrogenic and develops after caesarean section or open or laparoscopy surgeries for the treatment of gynecological diseases [6]. The lesion is usually subcutaneous tissue with or without extension to the fascia of the rectus abdominis. In very rare cases (our case) endometriosis can be detected individually in rectus abdominis [7]. Few such cases have been reported in the literature by when it was first described as a separate nosological entity by the Amato and Levitt in 1984 [8]. The metastatic theory, based on which endometrial cells reach extrapelvic areas through blood vessels or lymph system, seems to be able to largely explain the development of intrauterine focus at the level of the rectus abdominis [9] The time of appearance of symptomatic endometriosis foci in the rectus abdominis vary. In our case, it was 7 months after caesarean section. In bibliography states that it ranges from 1 to 24 years with an average of 4.8 years [10].

Despite the great development that has been achieved in recent years in imaging techniques, preoperative diagnosis is difficult and a challenge for gynecologists and surgeons. The diagnosis is usually made late and accidentally during gynecological surgery or postoperatively. Diagnosis is confirmed after histological examination of the surgical preparation [11]. The cyclical abdominal pain depending on menstruation and a history of caesarean section or gynecological surgery on a woman of childbearing age are the main ones clinical features that raise a serious suspicion of existence of endometriosis of the abdominal wall [9]. Unlike most cases described in the bibliography, in our case its absence of characteristic circular pain created additional difficulties in the preoperative diagnostic examination of the patient, resulting in chronic pelvic pain to most likely incorrectly attributed to the presence of uterine fibroids. In rare cases of endometriosis formation, abdominal pain is possible to get form of acute abdomen [12]. The difficulty and delay of diagnosis are attributed mainly in rarity of disease, but also in a wide range of pathological conditions, such as hematoma, granuloma, abscess, lipoma, lymphadenopathy, neuroma, sebaceous cyst, inguinal hernia, postoperative ventral hernias be included in its differential diagnosis endometriosis of the anterior abdominal wall [1].

Ultrasound is a first line tool in diagnostics approach to extrapelvic endometriosis. With abdominal ultrasound can be detected, intramuscular endometriosis in the anterior abdominal wall, the characteristics of which may vary from a completely solid mass or a mix echogenic mass with solid and cystic elements [13]. Also, the transvaginal ultrasound is useful in investigating pelvic structures, as it is estimated that 25% of women with extrapelvic endometriosis coexist with pelvic disease [14]. In addition, computed tomography and magnetic resonance imaging are available to provide valuable information on location, depth, extent and the spread of damage to adjacent tissues [15]. In our case, both transabdominal ultrasound and computed tomography and magnetic resonance imaging tomography were not able to diagnose the presence of ectopic endometriosis lesions at the level of the rectus abdominis. This fact delayed diagnosis of endometriosis of the muscular abdominal wall, further strengthening more the hypothesis that her uterine fibroids are responsible for chronic pelvic pain. Finally, a fine needle aspiration biopsy (FNA) can easily confirm the cytological diagnosis of ectopic endometriosis tissue and contribute to the most appropriate treatment planning of the disease [16].

The treatment of endometriosis of the rectus abdominis muscle requires surgery. The treatment of choice is wide local resection of the lesion on sound boundaries. The local exclusion of the lesion that can be done under ultrasound monitoring [17] is usually therapeutic and at the same time ensures confirmation of the diagnosis. Intraoperatively, care must be taken to avoid tissue abuse of the endometrial mass and for the careful cleaning of adjacent of the damage tissue in order to prevent reimplantation of microscopic disease residues and to minimize the possibility of recurrence [18]. In those cases where the gap is large, and the muscle filling is, the use of a grid is considered impossible [1]. Grid application was also done in our case. Drug therapy with LH - RH agonists progestogens or aromatase inhibitors appear to offer only temporary relief from symptoms [1]. Complementary medication after surgery should be considered in cases of suspected resection of the lesion on unhealthy margins, with, in order to prevent recurrence of the disease [19]. It was not recommended to our patient further therapeutic drug intervention, as it was considered to be complete excision of the endometrial focus from the muscular abdominal wall. The prognosis is usually good. Malignant mutation is very rare [20].

## Conclusion

Preoperative diagnosis of extrapelvic endometriosis with individual localization in abdominal muscles is a challenge in daily clinical practice. Despite the rarity of endometrial lesion in the muscular abdominal wall, the significant increase of the percentage of caesarean sections observed in recent years is necessary the inclusion of this rare extrapelvic form of endometriosis in the differential diagnosis between all the painful masses of the abdominal wall, thus allowing the early diagnosis and avoidance unnecessary diagnostic and therapeutic interventions. Early diagnosis and selection of the most appropriate therapeutic manipulations are judged currently necessary in order to minimize the risk of recurrence and to avoid the possibility of malignant recurrence of the disease.
